# Articles

**Published:** 1904-11

**Authors:** R. Ottolengui


New York, Oct. 28, 1904.

Editor of American Journal of Dental Science, Madison Wis.

Dear Sir:—

Permit me to take exception to the first article in your issue of October, under the title "International Dental Congress." This article you have credited to "Items of Interest," which would naturally lead your readers to believe that the entire article had first appeared in our magazine. This is a view which I am quite unwilling should be maintained. You have written the introduction to the article yourself, and you have a paragraph therein which is absolutely contrary to the facts. This paragraph reads as follows: "Dr. Rodrigues Ottolengui of New York placed in nomination for the same office Dr. C. N. Johnson of Chicago, but the latter withdrew." Dr. C. N. Johnson never withdrew because his name never was placed in nomination. I am the more exasperated by this statement because in my report of the Congress I very carefully abstained from entering into the unfortunate circumstances which surrounded the selection of officers at the Congress. I did so in the interests of the good name of American dentistry but I have facts in my possession with proof thereof which if printed would fully refute the idea that Dr. Burkhart was elected over Dr. Johnson and if there is any further publication of this misstatement, which has now appeared in two magazines, I give notice that I will publish all that I know.

Yours truly,

R. Ottolengui.

